# Content Analysis of Oncology-Related Pharmaceutical Advertising in a Peer-Reviewed Medical Journal

**DOI:** 10.1371/journal.pone.0044393

**Published:** 2012-08-31

**Authors:** Kan Yonemori, Akihiro Hirakawa, Masashi Ando, Taizo Hirata, Mayu Yunokawa, Chikako Shimizu, Kenji Tamura, Yasuhiro Fujiwara

**Affiliations:** 1 Breast and Medical Oncology Division, National Cancer Center Hospital, Tokyo, Japan; 2 Center for Advanced Medicine and Clinical Research, Nagoya University Graduate School of Medicine, Aichi, Japan; University of North Carolina at Chapel Hill, United States of America

## Abstract

**Background:**

The oncology market represents one of the largest pharmaceutical markets in any medical field, and printed advertising in medical journals is an important channel by which pharmaceutical companies communicate with healthcare professionals. The aim of the present study was to analyze the volume and content of and trends and changes in oncology-related advertising intended for healthcare professionals in a peer-reviewed medical journal. Information that could be included in advertisements to promote drug development and improve treatment strategies for cancer patients is discussed on the basis of the results of the analysis.

**Methods/Principal Findings:**

Overall, 6,720 advertisements covering 13,039 pages in a leading oncology medical journal published (by the American Society of Clinical Oncology) between January 2005 and December 2009 were analyzed. The advertisements targeting pharmaceuticals and clinical trials, in particular, were reviewed. A total of 6,720 advertisements covering 13,039 pages were included in the analysis. For the years 2005–2009, the percentages of total journal pages dedicated to advertising were 24.0%, 45.7%, 49.8%, 46.8%, and 49.8%, respectively. Package insert information and efficacy and safety explanations appeared in more than 80% of advertisements intended for pharmaceutical promotion. From 2005 to 2009, the overall quantity of drug advertisements decreased by approximately 13%, whereas advertisements calling for the enrollment of patients into registration trials increased by approximately 11%.

**Conclusion/Significance:**

Throughout the study period, oncology-related pharmaceutical advertisements occupied a considerable number of pages relative to other journal content. The proportion of advertisements on ongoing clinical trials increased progressively throughout the study period.

## Introduction

Most healthcare professionals are member of several medical societies, which publish scientific journals and literature to highlight the progress being made in that particular discipline. Owing to the broad readership of such journals including medical professionals, advertising in scientific journals is a powerful means of information distribution [1], [2].

Oncology is the field of medicine concerned with the diagnosis and treatment of cancer, a major causes of death in worldwide. This medical specialty attracts a high degree of interest from investigators, physicians, private industry, regulatory agencies, patients, and the general public. Many companies in the healthcare sector invest heavily in the development of novel pharmaceuticals and medical services related to oncology. Although it is best for oncology practice to proceed according to evidence from clinical trials and guidelines of scientific societies, advertising by private companies promotes products or services in order to gain advantages over competitors. Pharmaceuticals can be advertised through many types of avenues, among which medical journals are one of the most profitable [3]. To date, no summaries exist of the different types of advertising in oncology journals and the information that those advertisements provide.

In this study, the intentions of pharmaceutical companies were examined by analyzing the volume and content of and changes in oncology-related pharmaceutical advertising targeted at healthcare professionals in a peer-reviewed medical journal. We thought this investigation would suggest the role that pharmaceutical companies envision for advertising. To this end, we considered the advertising data published between 2005 and 2009 in the *Journal of Clinical Oncology* (JCO), which had a 2010 impact factor of 18.970 (ranking 4th among 184 oncology journals) and is published thrice monthly by the American Society of Clinical Oncology (ASCO) [4].

## Methods

For our analysis, we selected the JCO because ASCO is a representative scientific society on oncology, and its journal has a global readership among oncologists. Two experienced medical oncologists (KY and TH) assessed hard copies of the advertisements published in the JCO between January 2005 and December 2009, and any disagreements were resolved by consensus. The data analyzed included the numbers of journal and advertising pages, type of advertisements (pharmaceutical drugs, promotions, pharmaceutical approvals, medical device promotions and approvals, scientific meeting information, enrollment solicitations for trials, cancer support group information, medical system information, pharmaceutical company information, and other), and their content.

**Figure 1:**
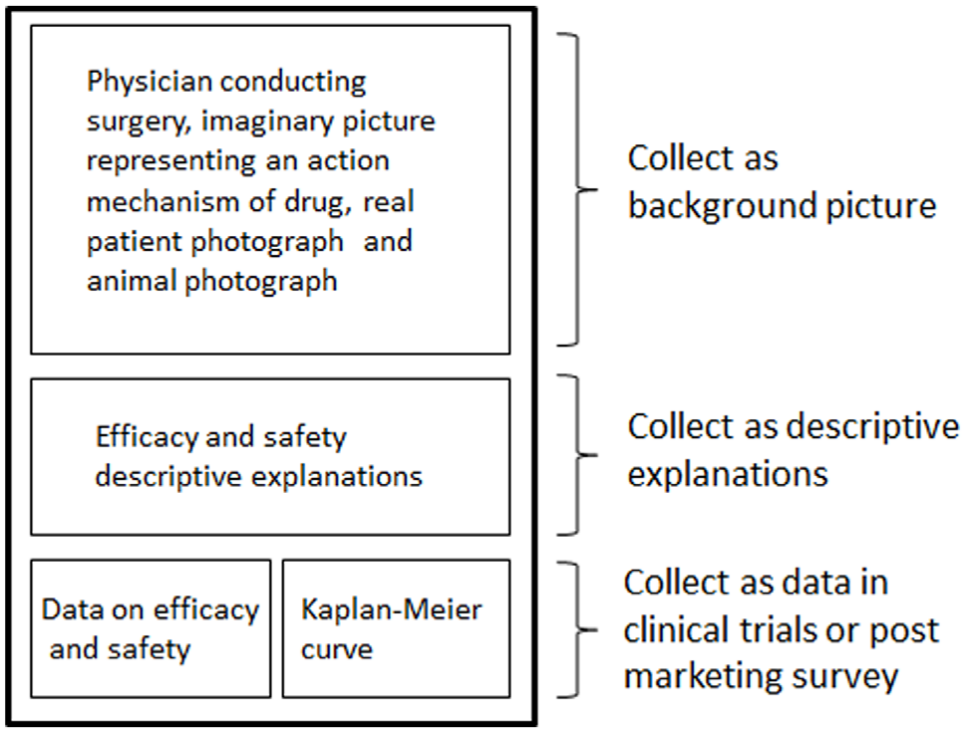
Example of the content of a typical print advertisement for a pharmaceutical drug and the data collection method.

For each pharmaceutical advertisement, the presence/absence of each of the following items was investigated: drug efficacy and safety, descriptive explanations of drugs, package insert information, data from clinical trials or post-marketing surveys (e.g., data on efficacy and/or safety, Kaplan–Meier curve for registration trials, or differences from other agents), and background images (e.g., pictures of patients, pictures of physicians, testimonials, biological images, product photographs, animal photographs, or diagrammatic representations of efficacy). Multiple categories were allowed for each element of the advertisements. We compared the characteristics and presentation material between advertisements for all drugs and those for 14 drugs that received accelerated approval from the Food and Drug Administration (FDA). Figure 1 shows an example of the content of a typical advertisement for a specific drug and the data collection method used. The data listed above were collected for each advertisement, as shown in Figure 1. For advertisements calling for enrollment in trials, the information analyzed included an explanation of trial design (randomized, blind, or multiregional), trial summary, endpoints (primary and secondary), sample size, mechanism of action of the investigational agent, the clinical trial registration numbers, main eligibility criteria, and cautionary statements.

## Results

The descriptive statistics of advertisements in each year of the study period are shown in Table 1. According to Table 1, the proportion of advertisements intended for pharmaceutical promotion decreased by 13% during the sampling period, whereas calls for the enrollment of patients in continuous-registration clinical trials increased by 11%.

**Table 1:** Quantity and content of advertisements by year.

Year		2005	2006	2007	2008	2009
Advertisements	Total journal pages	9445	5790	5845	6021	4819
	Total advertising pages	2264	2647	2909.75	2819.5	2398.6
	Proportion of advertising pages, %	24.0	45.7	49.8	46.8	49.8
	Mean pages per advertisement (SD)	1.9 (1.6)	2.2 (1.9)	1.9 (1.9)	1.9 (2.1)	1.8 (1.6)
	Number of advertisements, n	1177	1208	1538	1469	1328
Content	Pharmaceutical promotion, n (%)	535 (45.5)	560 (46.4)	641 (41.7)	468 (31.9)	432 (32.5)
	Announcement of pharmaceutical approval, n (%)	33 (2.8)	42 (3.5)	38 (2.5)	46 (3.1)	30 (2.3)
	Medical device promotion, n (%)	20 (1.7)	25 (2.1)	30 (2.0)	22 (1.5)	21 (1.6)
	Announcement of medical device approval, n (%)	0 (0.0)	0 (0.0)	3 (0.2)	0 (0.0)	0 (0.0)
	Scientific meeting information, n (%)	87 (7.4)	127 (10.5)	193 (12.6)	177 (12.1)	136 (10.2)
	Calls for the enrollment of patients in registrationtrials, n (%)	54 (4.6)	44 (3.6)	134 (8.7)	236 (16.1)	206 (15.5)
	Cancer support group information, n (%)	27 (2.3)	28 (2.3)	32 (2.1)	36 (2.5)	48 (3.6)
	Medical support system information, n (%)	0 (0.0)	5 (0.4)	25 (1.6)	36 (2.5)	71 (5.4)
	Pharmaceutical company information, n (%)	54 (4.6)	94 (7.8)	22 (1.4)	56 (3.8)	65 (4.9)
	Other, n (%)	367 (31.2)	283 (23.4)	420 (27.3)	392 (26.7)	319 (24.0)

Table 2 shows the descriptive statistics of pharmaceutical advertisements. Brief package insert information and efficacy and safety explanations extracted from approved package inserts appeared in more than 80% of the advertisements. During the 5 years included in the study, the use of pictures and photographs decreased, and inclusion of the results of clinical trials increased. The results encompassing advertisements for all drugs in Table 2 and the results regarding drugs that received accelerated approval showed similar patterns (data not shown).

**Table 2:** Characteristics of and presentation material in advertisements promoting pharmaceuticals by year.

Content	Year	2005	2006	2007	2008	2009
Advertisements	Total number	535	560	641	468	432
	Mean number of pages per advertisement	2.8	3	2.9	3.3	3.3
	(SD)	(1.4)	(1.5)	(1.8)	(1.7)	(1.6)
	Minimum and maximum numbers of pages	1–9	1–10	0.5–11	0.25–11.0	0.25–7.0
	Brief package insert information*, n (%)	475 (88.8)	508 (90.7)	529 (82.5)	416 (88.9)	385 (89.1)
Descriptiveexplanation	Efficacy, n (%)	418 (78.1)	468 (83.6)	415 (64.7)	332 (70.9)	323 (74.8)
	Safety, n (%)	471 (88.0)	511 (91.3)	551 (86.0)	400 (85.5)	379 (87.7)
Data in clinical trials orpost-marketing survey	Efficacy and/or safety data, n (%)	64 (12.0)	102 (18.2)	353 (55.1)	321 (68.6)	318 (73.6)
	Kaplan–Meier curve, n (%)	66 (12.3)	95 (17.0)	113 (17.6)	157 (33.6)	132 (30.6)
	Differences from other agents (including placebo), n (%)	240 (44.9)	268 (47.9)	249 (38.9)	241 (51.5)	223 (51.6)
Backgroundpictures	Real patient photograph, n (%)	46 (8.6)	25 (4.5)	11 (1.7)	7 (1.5)	13 (3.0)
	Patient images, n (%)	206 (38.5)	219 (39.1)	258 (40.3)	133 (28.4)	109 (25.2)
	Physician images, n (%)	1 (0.2)	6 (1.1)	30 (4.7)	20 (4.3)	36 (8.3)
	Biological images, n (%)	171 (32.0)	174 (31.1)	90 (14.0)	24 (5.1)	22 (5.1)
	Product photographs, n (%)	116 (21.7)	95 (17.0)	38 (5.9)	36 (7.7)	66 (15.3)
	Animal photographs, n (%)	65 (12.2)	37 (6.6)	32 (5.0)	23 (4.9)	28 (6.5)
	Illustrations representing efficacy, n (%)	54 (10.1)	34 (6.1)	117 (18.3)	106 (22.7)	156 (36.1)

* Brief summary is extracted from approved package insert.

Table 3 shows the descriptive statics of advertisements soliciting participation in trials. The proportion of advertisements calling for the enrollment of patients into phase III registration trials increased throughout the study period. In addition, the explanation of trial design increased over time.

**Table 3:** Information provided in advertisements for trial registration by year.

Year		2005	2006	2007	2008	2009
Advertisements	Total number	54	44	134	236	206
	Mean number of pages per advertisement	1.0	1	1.1	1.2	1.0
	(SD)	(0.1)	–	(0.3)	(0.4)	(0.1)
	Minimum and maximum numbers of pages	1.0 to 2.0	1 to 1	0.5 to 2.0	0.5 to 2.0	0.5 to 2.0
Calls for enrollment ofpatients in registration trials	Phase I, n (%)	0 (0.0)	0 (0.0)	5 (3.7)	0 (0.0)	0 (0.0)
	Phase II, n (%)	21 (38.9)	15 (34.1)	42 (31.3)	29 (12.3)	46 (22.3)
	Phase III, n (%)	16 (29.6)	14 (31.8)	77 (57.5)	201 (85.2)	150 (72.8)
	Unknown, n (%)	17 (31.5)	15 (34.1)	10 ′7.5)	6 (2.5)	10 (4.9)
Explanation of trial design	Randomization, n (%)	18 (33.3)	17 (38.6)	79 (59.0)	218 (92.4)	158 (76.7)
	Blinding, n (%)	14 (25.9)	12 (27.3)	64 (47.8)	138 (58.5)	75 (36.4)
	Multiregionality, n (%)	2 (3.7)	0 (0.0)	41 (30.6)	124 (52.5)	56 (27.2)
	Brief summary of trial, n (%)	28 (51.9)	16 (36.4)	104 (77.6)	188 (79.7)	145 (70.4)
	Primary endpoint, n (%)	1 (1.9)	16 (36.4)	109 (81.3)	199 (84.3)	138 (67.0)
	Secondary endpoint, n (%)	1 (1.9)	9 (20.5)	32 (23.9)	28 (11.9)	57 (27.7)
	Sample size, n (%)	5 (9.3)	9 (20.5)	37 (27.6)	127 (53.8)	105 (51.0)
	Mechanism of action of investigationalagent, n (%)	1 (1.9)	0 (.0)	25 (18.7)	38 (16.1)	56 (27.2)
	Code number of clinical trialregistration, n (%)	6 (11.1)	9 (20.5)	19 (14.2)	42 (17.8)	74 (35.9)
	Main eligibility criteria	49 (90.7)	36 (81.8)	119 (88.8)	210 (89.0)	127 (61.7)
	Cautionary statement of disapprovedindication, n (%)	0 (0.0)	0 (0.0)	89 (66.4)	173 (73.3)	134 (65.1)

## Discussion

This is the first study to assess the nature and extent of oncology-related pharmaceutical advertising targeted at healthcare professionals and published in a leading oncology journal. The presentation of background material in advertising has shifted from patient or biological images to actual data and illustrations representing the efficacy of products. Practical information regarding enrollment in trials is currently provided in advertisements promoting trial participation.

In general, advertising for pharmaceutical products is subject to regulation and control. The International Federation of Pharmaceutical Manufacturers Association (IFPMA) established the *IFPMA Code of Pharmaceutical Marketing Practices* in 1981 [5]. The World Health Organization issued its *Ethical Criteria for Medicinal Drug Promotion* in 1988 [6]. These documents contain general principles for ethical advertising that are voluntary without regulatory or legal weight. Therefore, the laws and regulations of national drug regulatory authorities are the most important for accurate and not misleading advertising. In this study, the US FDA was the governing authority. A systematic review conducted on the years 1990 and 2005 indicated that pharmaceutical advertising in medical or pharmaceutical journals often provides poor quality information [7]. In addition, one study revealed that few physician-directed printed pharmaceutical advertisements adhere to all FDA guidelines; over half fail to quantify serious risks [8]. Generally, such advertising should obey rules governing its integrity, accuracy, clarity, and completeness in order to ensure that healthcare professionals are well informed about the currently available medicines and their applications. The present study selected JCO, which is an informative case because United States of America (US)-based medical journals and prescription drug advertising are under the control of the US FDA [9]. Continuous active monitoring and administration through the Office of Prescription Drug Promotion and initiatives such as the “Bad Ad” program that use assistance from advertising recipients may serve to improve the quality of information provided in healthcare professional-directed drug advertising [10].

Some slight differences exist regarding local regulations on drug advertising, such as prohibited target populations for prescription drug advertising. Basic local regulatory requirements surrounding the provision of core and appropriately balanced information in prescription drug advertising intended for medical professionals are similar between the US, the European Union, and Japan [9], [11], [12]. The present results indicate that most of the oncology related advertising by pharmaceutical firms during the period analyzed consisted of explanations of the efficacy and safety of products based on scientific research and package inserts. This information most often included Kaplan–Meier curves and data on the differences between the particular agent featured in the advertisement and other agents or a placebo rather than images of patients, drugs, animals, physicians, or biological processes.

The present study revealed that most drug advertising presented descriptions about efficacy and safety and information from package inserts in the limited space occupied by the advertisements. A previous study indicated that cardiac toxicity was underestimated in cancer-related clinical trials compared with FDA package inserts; however, this went widely unnoticed by many practitioners, likely because of the fact that few practitioners comprehensively read package inserts for the drugs they prescribe [13]. The package insert also repeatedly revises information, especially toxicity information gathered from larger samples, such as additional registration trial data from supplemental new drug applications or post-marketing surveys. Package insert and review information is free and has been noted as the most complete and objective form of safety and efficacy information because regulatory agencies carefully review and approve package insert revisions submitted by manufacturers [14]; this information could be utilized in appropriate drug advertising.

The recruitment of participants for trials can be extremely difficult. Our results showed that solicitations for patient enrollment in clinical trials increased between 2005 and 2009, with most of these being phase III, multiregional, and registration trials. Notably, the increasing number of advertisements for patient enrollment could be attributable to an increase in the overall number of clinical trials being conducted. On the other hand, the present study showed that advertisements calling for the enrollment of patients in ongoing registration trials provided practical information of trial design to healthcare professionals. Effective strategies for improving recruitment could be of great benefit to researchers designing and running trials [15]. Multinational collaborative groups supported by pharmaceutical companies advocated for global phase III trials in order to accelerate the development of oncology drugs [16]. Currently, no verified method exists to increase the participation of cancer patients in randomized trials [17]. The present study showed a dramatic increase in cautionary statements in advertising for ongoing registration trials. The reason for this increase is unknown; however, the provision of appropriate information about an unapproved drug is preferable for adherence to local regulations. The efficacy of advertisements in medical journals for healthcare professionals is unknown, and further research is required to evaluate the effects of advertising in medical journals on levels of patient participation in trials.

The present study has several limitations. First, the study included only a single journal, JCO. Sieber et al. reported that JCO had the most advertising of any journal in the field of hematology/oncology in 2006 [18]. Although it is the leading oncology journal, it is not representative of all important medical journals. The fact that the present study analyzed longitudinal data from only one oncology journal could be a source of bias. Future studies could include additional journals and other forms of media and employ longitudinal and cross-sectional designs. Admittedly, the collected information and its categorization (Tables 1–3) may be somewhat arbitrary, although 2 experienced medical oncologists assessed all the advertisements. Concerns over rising drug costs, pharmaceutical advertising, and potential conflicts of interest have focused attention on physician prescribing behavior [19]. Further studies are needed to analyze the influence of advertisements on decision making and the behavior of healthcare professionals. Further assignment of advertisement attributes would be helpful in order to analyze the adherence vs. non-adherence of oncology drug and registration trial advertising to FDA guidelines. Pharmaceuticals and medical devices are commercial products that are inescapably tied to advertising; however, advertising must take place within rational limits. The International Committee of Medical Journal Editors (ICMJE) lists the requirements for publishing and editorial issues related to publication in biomedical journals [20]. According to the statement of the ICMJE, advertising should not be allowed to influence editorial decisions, and journals need formal, explicit written policies for advertising in both the print and electronic versions of their journals. The present study only analyzed advertising in printed media. The use of the internet as a resource for health-related information, including advertising, is likely to increase and play an important role. In the future, collaborative regulatory systems for pharmaceutical and medical devices need to maintain the quality of information available [21].
